# Supplementary material for: A Competitive “On-Off-Enhanced On” AIE Fluorescence Switch for Detecting Biothiols Based on Hg2+ Ions and Gold Nanoclusters
Source: Biosensors (Basel). 2022 Dec 27;13(1):35. doi: 10.3390/bios13010035 (PMC9856123; doi:10.3390/bios13010035)
Supplement: Supplementary file 1 [file biosensors-13-00035-s001.zip › biosensors-2083372-supplementary.pdf]

Supporting information

A Competitive "On-Off-Enhanced On" AIE Fluorescence Switch for Detecting Biothiols Based on Hg^{2+} Ions and Gold Nanoclusters

Shuqi Li [1,2,†], Yuqi Wan [1,2,†], Yu Li [1,2], Jinghan Liu [1,2], Fuwei Pi [1,2,*] and Ling Liu [3,*]

1 State Key Laboratory of Food Science and Technology, School of Food Science and Technology, Jiangnan University, Wuxi 214122, China; lsq14124869@163.com (S.L.); yuqiwan07@163.com (Y.W.); m18015366421@163.com (Y.L.); jinghanliu55@foxmail.com (J.L.)

2 Collaborative Innovation Center of Food Safety and Quality Control in Jiangsu Province,
Jiangnan University, Wuxi 214122, China

3 Wuxi Institute of Technology, Wuxi 214122, China

* Correspondence: pifuwei@jiangnan.edu.cn (F.P.); liulin@wxit.edu.cn (L.L.);

† These authors contributed equally to this work.

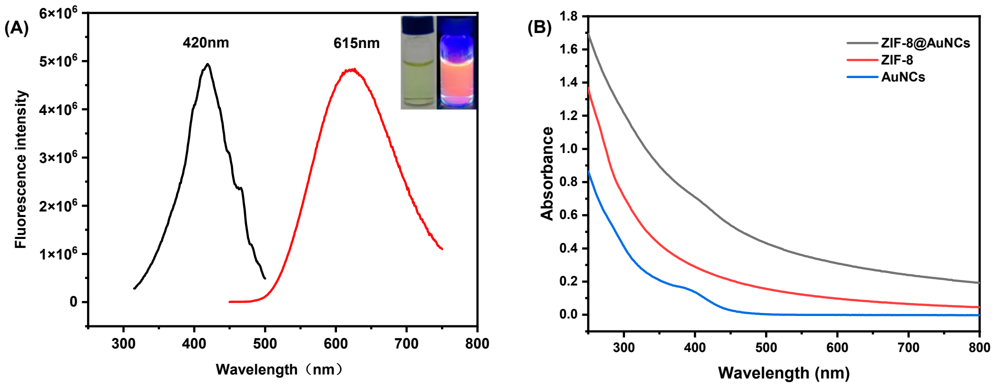

Figure S1. (A) Fluorescence excitation and emission spectra of as-prepared GSH-AuNCs. (B) UV-vis spectra of ZIF-8, AuNCs and AuNCs/ZIF-8

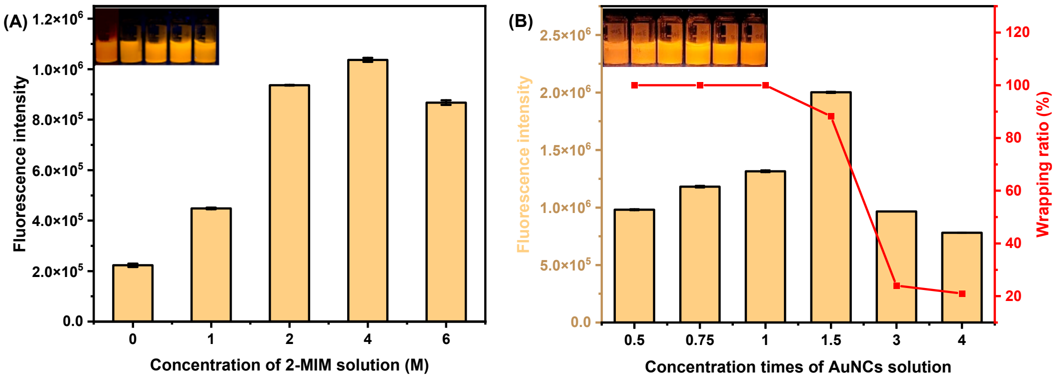

Figure S2. The fluorescence emission spectra of AuNCs/ZIF-8 synthesized from (A) different concentration of 2-MIM solution and (B) different concentration times of AuNCs solution. The red broken line represents the coverage of AuNCs.

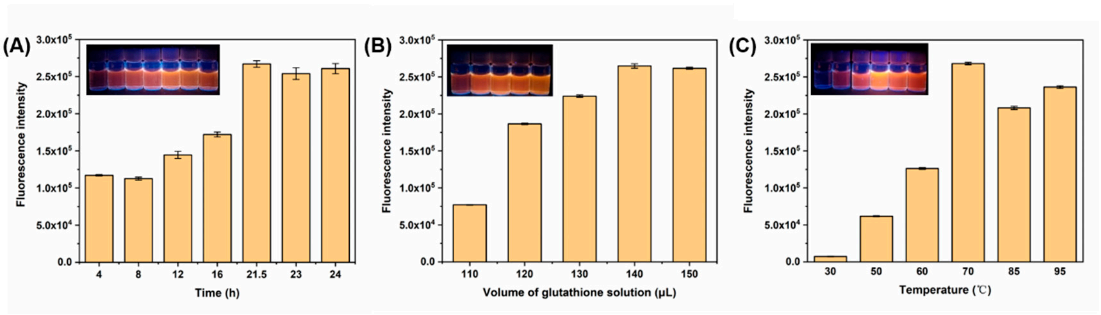

Figure S3. The effect of (A) synthesis time (B) Volume of GSH solution and (C) synthetic temperature on the fluorescence intensity of AuNCs.
